# Consumption in Cows

**Published:** 1881-07

**Authors:** 


### CONSUMPTION IN COWS.

On the authority of the *London Medical Record*, Dr. Heath, president of the American Farmer's Club, recently read a very important paper before that society on the tuberculosis in domestic animals, and some of its effects on human health. He says that this disease prevails extensively among such animals all over the world, and especially in populous and crowded localities. Cows which are kept shut up in close, foul air, as is the case with large numbers in and about London, are very liable to it. He says that observations in Mexico have led to the conclusion that thirty-four per cent. of all beasts slaughtered there are more or less affected with this disease, and he is of the opinion that fifty per cent. of the cows kept in large towns are thus diseased. The fact that this is not more generally recognized is of course owing to the animals being slaughtered before the disorder has attained any very noticeable development. According to Dr. Heath, if cows, like human beings, were allowed to die from natural causes, the proportion succumbing to tuberculosis would be quite as great, and probably much greater.

